# First Draft of Agreed Bill

**Published:** 1916-06-17

**Authors:** 


### THE REGISTRATION OF NURSES.

### First Draft of Agreed Bill.

### A BILL TO PROVIDE FOR THE REGISTRATION OF NURSES (6 & 7 GEO. 5), A.D. 1916.

Be it enacted by the King's most Excellent Majesty, by and with the advice and consent of the Lords Spiritual and Temporal, and Commons, in this present Parliament assembled, and by the authority of the same as follows:—

1. Short Title.—This Act may for all purposes be cited as the Nurses' Registration Act, 1916.

2. The College of Nursing.—(1) The College of Nursing, Limited, shall be entitled to bear the title of the General Nursing Council and College of Nursing without the addition of the word "Limited," and is hereinafter described as the General Nursing Council and College of Nursing.

(2) Every nurse registered under this Act shall be entitled to a vote at elections of the Council, and without further fee to become a member of the College of Nursing.

3. Register of Nurses.—It shall be the duty of the College of Nursing to form and to keep a Register of Nurses and (if they think fit) a Register of Mental Nurses, and each such Register is hereinafter in this Act included in the term "the Register." The Register already formed by the College of Nursing shall be the first Register under this Act. The College of Nursing shall for the purposes of this Act act by the Council of the College as regulated by rules made under this Act.

4. Rules.—(1) Rules may be made under this Act—

(i) regulating the constitution and proceedings of the Council of the College of Nursing and providing, if thought fit, for the representation thereon of the Privy Council and any Government Department, and of the medical profession. Provided that not less than two-thirds of the Council shall be elected by the members of the College of Nursing;

(ii) regulating the issue and cancellation of certificates of registration and the conditions of admission to the Register;

(iii) regulating the course of training and the examination of nurses intending to be registered and the appointment of examiners;

(iv) regulating the admission to the Register of persons already in practice as trained nurses at the passing of this Act;

(v) regulating the admission to the Register of persons registered in any British Possession in which a Nurses' Registration Act is in force subject to such conditions and qualifications as the rules may prescribe;

(vi) providing for the publication of the Register with such particulars relating to the qualification of the nurses as the rules may prescribe;

(vii) providing for the temporary or permanent removal from the Register of any nurse for such causes or offences and after such enquiry as the rules may prescribe;

(viii) regulating the restoration to the Register of any nurse so removed if the College of Nursing thinks fit;

(ix) providing for the constitution and regulating the powers of committee for any parts of the United Kingdom for the purposes of this Act; and

(x) otherwise for the purposes of this Act.

(2) Rules under this Act shall be made by the Council, but shall have no effect until they have been approved by the Privy Council, and the Privy Council may approve the rules subject to such modification as the Privy Council think proper.

5. Council.—On the passing of this Act, the first Council shall be appointed as to one-third by the Privy Council and/or any Government Department, and/or by the medical profession; as to one-third by the Council of the College of Nursing, Limited, and as to one-third by the Central Committee for the State Registration of Trained Nurses, and shall hold office for one year.

6. Fees.—Every candidate for examination or registration shall pay to the College of Nursing such fee as may be prescribed by the rules.

7. Penalty for Pretending to be Registered.—From and after the publication of the first Register no person shall be entitled to take or use the name or title of registered nurse, or of registered mental nurse (either alone or in combination with any other word or words or letters), or any name, title, addition, or description implying that he or she is registered under this Act, or is recognised by law as a registered nurse, or as a registered mental nurse, unless that person is registered as such under this Act, and, if any person knowingly takes or uses any such name, title, addition, or description in contravention of this section, he or she shall be liable on summary conviction to a fine not exceeding ten pounds.

8. Copy of Register to be Evidence.—A copy of the Register certified to be a true copy by the secretary of the College of Nursing shall be evidence in all Courts of Law that nurses whose names are therein specified are registered under this Act, and the absence of the name of any nurse from the Register shall be evidence that such nurse is not registered under this Act;

Provided always, that in the case of any nurse whose name does not appear in the Register a certificate under the hand of the secretary that the name of such nurse has been entered on the Register shall be evidence that such nurse has been duly registered under this Act.

9. Penalty for Obtaining Certificate by False Representation and for Falsification of Register.—Any person—(1) who procures or attempts to procure a certificate under this Act by making or causing to be made or produced any false and fraudulent declaration, certificate, or representation, either in writing or otherwise; or (2) who wilfully makes or causes to be made any falsification in any manner relating to the Register, shall be guilty of a misdemeanour, and shall on conviction thereof be liable to be imprisoned, with or without hard labour, for any term not exceeding twelve months.

10. Provision as to Prosecutions.—A prosecution for any of the offences in this Act mentioned shall not be instituted by a private person without the consent of the College of Nursing, but with such consent may be instituted by the College of Nursing.

11. No Authority to Practise Medicine.—Nothing contained in this Act or in any rules made thereunder shall confer any authority to practise medicine, or to undertake the treatment or cure of disease.

12. Laying of Rules Before Parliament.—Rules under this Act shall be laid before both Houses of Parliament as soon as may be after they have been approved by the Privy Council.

